# Occurrence and assemblage composition of millipedes (Myriapoda, Diplopoda) and terrestrial isopods (Crustacea, Isopoda, Oniscidea) in urban areas of Switzerland

**DOI:** 10.3897/zookeys.176.2153

**Published:** 2012-03-20

**Authors:** Ferenc Vilisics, Dávid Bogyó, Thomas Sattler, Marco Moretti

**Affiliations:** 1 University of Helsinki, Faculty of Biological and Environmental Science, Department of Environmental Science, 00014 Helsinki, Viikinkaari 2, Finland; 2 Department of Ecology, University of Debrecen, H-4010 Debrecen, PO Box 71, Hungary; 3 Swiss Federal Research Institute WSL, Community Ecology Research Unit, Via Belsoggiorno 22, 6500 Bellinzona, Switzerland

**Keywords:** Decomposers, urbanization, woodlice, urban biodiversity, arthropods

## Abstract

Terrestrial isopods and millipedes, members of the invertebrate macro-decomposer guild, were collected through pitfall traps in three Swiss cities (Zurich, Lucerne, Lugano). A total of 7,198 individuals of 17 isopod species (7093 ind.), and 10 millipede species (105 ind.) were captured. Besides the Alpine endemic isopod (*Trichoniscus alemannicus*) and millipede (*Cylindroiulus verhoeffi*), urban assemblages were mainly composed of widespread, native European and even cosmopolitan species, which are frequent in anthropogenic areas. Overall species richness (isopods and millipedes combined) was similar in Zurich (17 species) and Lucerne (16), while only 13 species were sampled in Lugano. According to the Sørensen index of similarity, species composition of Zurich and Lucerne were more alike, while the one of Lugano was more distinct from the other two cities. This result can be explained by the spatial proximity of Zurich and Lucerne in the north of the Alps compared to Lugano, which is located more distantly and in the south of the Alps. Dominant isopods and millipedes in Zurich and Lucerne were found to be widespread synanthropic species in temperate Europe(*Porcellio scaber*, *Trachelipus rathkii* and *Ophyiulus pilosus*) while the dominant isopod in Lugano (*Trachelipus razzautii*) is a species with a north-eastern Mediterranean distribution. Our study reveals that the urban millipede and isopod fauna in Swiss cities mainly consists of widespread species, but species of narrower distribution (e.g. *Trichoniscus alemannicus*, *Cylindroiulus verhoeffi*) may also find suitable habitats in cities. Despite some signs of biotic homogenization, our study also found compositional differences of millipede and isopod assemblages between northern and southern cities that suggest geographical effects of the regional species pool.

## Introduction

As one of the major factors of global change, urbanization and its effects on biodiversity have attracted great scientific attention in the past decade (e.g. McDonnell and Hahs 2008, Niemelä et al. 2000). Numerous studies have added an increasing knowledge to the understanding of the ecology of many taxa in urban environments, such as plants (e.g. Walker et al. 2009, Gulezian and Nyberg 2010), insects (e.g. Magura et al. 2004, Sattler et al. 2010a, 2010b, 2011), spiders (e.g. Magura et al. 2010), and vertebrates (e.g. Melles et al. 2003, Tóth et al. 2009, Fontana et al. 2011).

Urban soil meso- and macro-arthropods have received less attention (but see Korsós et al. 2002, Vilisics et al. 2007), despite their importance in ecosystem processes such as decomposition of organic matter (Hassall et al. 1987). Decay of dead plant matter results in ions readily available to uptake for plants. The majority of fallen leaves and woody debris are broken down by microbes, fungi and invertebrates (Berg and McClaugherty 2003).

Soil macro- meso- and micro-invertebrates contribute in the decomposition cascade by either fragmenting, or further mineralizing dead plant matter (Verhoef and Brussaard 1990). Key organisms in initial breakdown and comminution of dead matter are isopods, millipedes, termites, ants, and members of other invertebrate groups (Coleman and Hendrix 2000), while nematodes and annelids are essential for mineralization (Verhoef and Brussaard 1990, Flegel and Schrader 2000). Thus, millipedes (Myriapoda: Diplopoda) and isopods (Crustacea, Isopoda: Oniscidea) belong to the same functional guild, even though they are taxonomically quite distant (Scheu and Falca 2000). Based on this common ecosystem function, we propose to pool the two taxa in the same analyses which, to the best of our knowledge, is a rather novel approach in soil zoology and ecology.

Millipedes and isopods are known to inhabit European urban habitats, mainly by cosmopolitan and Holarctic species (Smith et al. 2006, Riedel et al. 2009, Vilisics and Hornung 2009). Human activities, such as gardening, transportation of soil, and cultivation of ornamental plants, are suspected to be the most important factors for species exchanges of less mobile organisms such as millipedes and isopods between distant locations. At the same time other studies (Bogyó and Korsós 2009, Riedel et al. 2009) have shown that also the native species pool has an effect on urban soil fauna, providing substrate for native species typical in natural and semi-natural habitats in cities. Particular urban habitats, such as botanical and private gardens and parks, however, harbour established populations of alien soil arthropods too (Vilisics and Hornung 2009, Cochard et al. 2010).

Urban assemblages of soil invertebrates show controversial patterns of species composition: some studies suggest that species compositions differ along urbanization gradients (Korsós 1992, Korsós et al. 2002, Bogyó and Korsós 2009, Riedel et al. 2009), while others have shown no differences along similar gradients (Hornung et al. 2007, Vilisics et al. 2007). In this study we analyse the occurrence and assemblage composition of urban millipede and isopod faunas in three urban areas of Switzerland. We discuss the most interesting findings with respect to European soil fauna. This contribution is part of the BiodiverCity project (www.biodivercity.ch) that aims to assess biodiversity in urban environments and its acceptance by citizens in the framework of the Swiss national research program ‘Sustainable development of the built environment’.

## Methods

### Study sites

The study took place in three Swiss cities, namely Zurich (371,000 inhabitants /92 km^2^), Lucerne (59,000 /24 km^2^) and Lugano (49,000/ 26 km^2^), which represent small to medium sized cities in central Europe. The cities studied lay on a north to south gradient (approx. 200 km, with Lugano south of the Alps) and all are bordered by a lake and mountains > 800 m. Originally, 36 sampling sites were selected in each city but at the end only 106 could be used for the analyses: 36 in Zurich and Lugano, 34 in Lucerne.

The three cities share common features such as historical centres, residential areas, business quarters, public green areas, parks and cemeteries, and former industrial areas. The cities are characterized by moderate temperature (North: average January temperature 1°C, July 17°C; South: January 3°C, July 20°C) with an annual precipitation of 1000 mm for Zurich, 1150 mm for Lucerne and 1600 mm for Lugano.

Within each of the three cities sampling points were selected along a continuous urbanization gradient, which was measured as the fraction of sealed and built area in the 50 m radius around the sampling points. The selection of the individual sampling points followed a reasoned choice sampling strategy to cover the entire urbanisation gradient (3% to 92% sealed and built area). We included a wide range of urban habitat types (private gardens, semi-public spaces of apartment buildings, public parks and courtyards of industrial buildings) into the study. Mean distance between study sites was 388 m (± 21 m SE). A minimal distance of 250 metres was kept between sampling sites and the city fringe. Precise locations of the study sites are given in Germann et al. (2008).

### Data collection

Isopods and millipedes were sampled through pitfall traps, consisting of 3 plastic cups (opening diameter 75 mm) per trap site recessed into the soil and arranged in an isosceles triangle with a distance of one meter. Transparent roofs installed approximately 8 cm above the cups provided protection from rain. Traps were emptied weekly during 7 weeks from June 13^th^ to August 3^rd^ 2006 (Sattler et al. 2010a), which corresponds to the period with highest arthropod activity in Switzerland (Duelli et al. 1999, Obrist and Duelli 2010).

Identification of millipedes was based on Schubart (1934) and Blower (1985). For isopod identification we used the keys of Schmölzer (1965) and Gruner (1966). Valid nomenclature was applied according to Schmalfuss (2003) and Enghoff (2010). The reference collection is deposited at the Natural History Museum in Lugano (Switzerland).

### Data analyses

We used species richness (number of species) as the most common measure to quantify biodiversity (Magurran 2004). Incidence is the frequency with which the species occurs at all in the study sites of a city. This value was used to assess steadiness of a species in the three cities. This value is indicative to the regularity of a species’ occurrence which does not necessarily correlate with abundance. We regarded incidence rate ‘high’ when it was over 50%, and ‘low’ when it did not reach 10%.

The similarity between the millipede and isopod species assemblages combined sampled in the three cities was assessed using the Sørensen index (Sørensen 1948), which is a widely used index in ecology and thus suitable for comparative purposes. For these analyses we used data of specimens identified to species level only.

## Results

### Species richness and composition

Overall, 17 species of isopods (7015 individuals) and 8 species millipedes (98 ind.) were identified in the three studied Swiss cities; one isopod could only be identified at genus level, while two millipedes only at family level.

**Figure 1.**
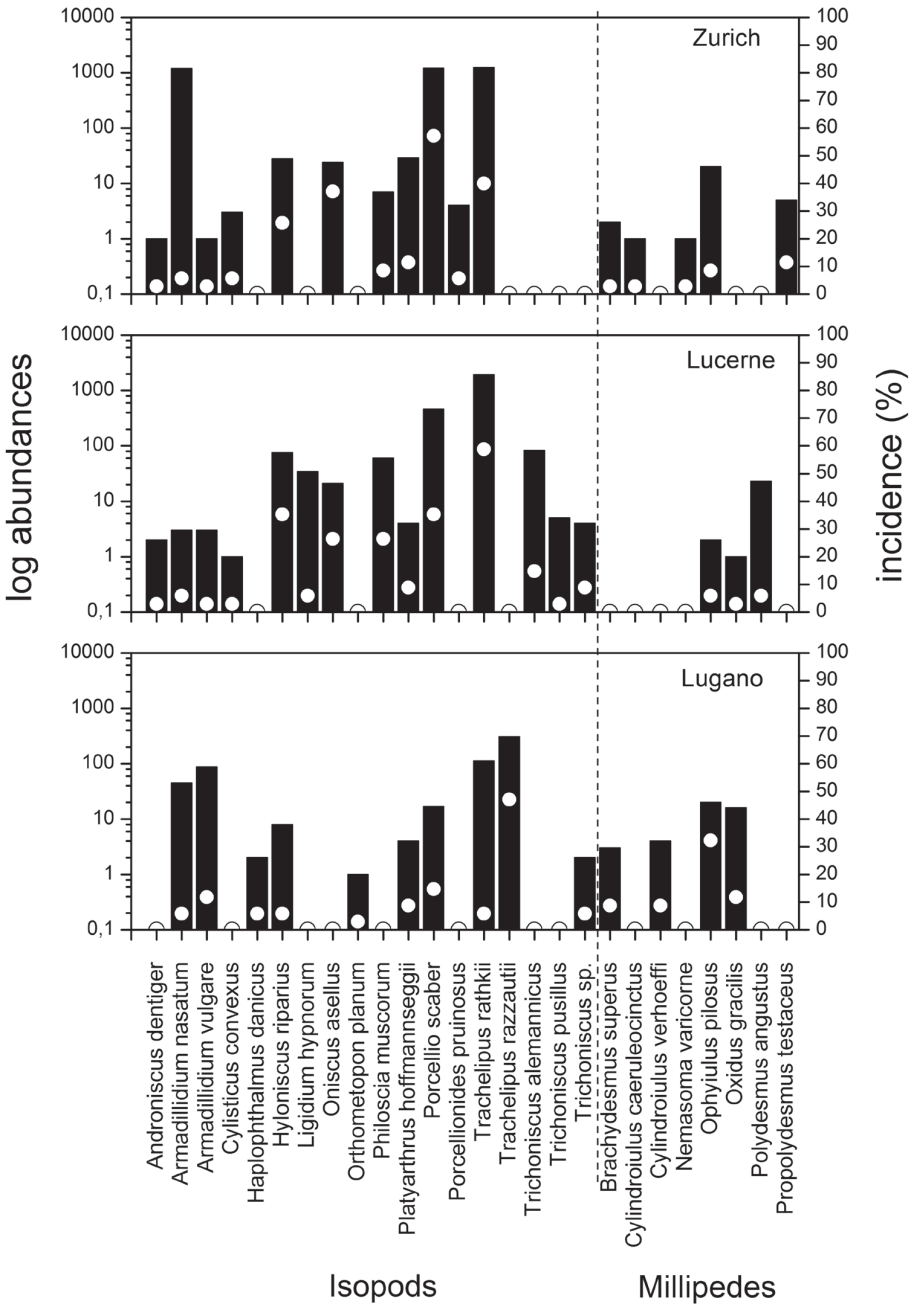
Abundances of isopods and millipedes in Zurich, Lucerne and Lugano in logarithmic scale. Dashed line separates isopods (on the left) from millipedes. Black bars represent abundances, white circles show the species’ incidences per all sites per city.

Isopod species richness was highest in Lucerne (14 species) and lowest in Lugano (10 species), with 11 species in Zurich (Table 1). One third (6 species) of all Isopoda species occurred in all three cities. Five additional species were captured in both Lucerne and Zurich but not in Lugano (Table 1). Three isopod species were dominant in the three cities (Fig. 1), i.e. the cosmopolitan *Porcellio scaber* Latreille, 1804 in Zurich (1216 individuals, 32% relative abundance); the widespread European *Trachelipus rathkii* (Brandt, 1833)in Zurich (1934, 33%) and Lucerne (1234, 72%), and the Mediterranean *Trachelipus razzautii* (Arcangeli, 1913) in Lugano (307, 52.3%).

**Table 1.** Incidence of isopod and millipede species in sampling sites in the cities of Zurich, Lucerne, and Lugano.

**Species**	**Species occurrences in traps**		
	**Zurich (n=36)**	**Lucerne (n=34)**	**Lugano (n=36)**
*Androniscus dentiger* Verhoeff, 1908	+	+	0
*Armadillidium nasatum* Budde-Lund, 1885	+	+	+
*Armadillidium vulgare* (Latreille, 1804)	+	+	+
*Cylisticus convexus* (De Geer, 1778)	+	+	0
*Haplophthalmus danicus* Budde-Lund, 1880	0	0	+
*Hyloniscus riparius* (C. Koch, 1838)	+!	+!	+
*Ligidium hypnorum* (Cuvier, 1792)	0	+	0
*Oniscus asellus* Linnaeus, 1758	+!	+!	0
*Orthometopon planum* (Budde-Lund, 1885)	0	0	+
*Philoscia muscorum* (Scopoli, 1763)	+	+!	0
*Platyarthrus hoffmannseggii* (Budde-Lund, 1893)*	+	+	+
*Porcellio scaber* Latreille, 1704	+!	+!	+
*Porcellionides pruinosus* (Brandt, 1833)	+	0	0
*Trachelipus rathkii* (Brandt, 1833)	+!	+!	+
*Trachelipus razzautii* (Arcangeli, 1913)	0	0	+!
*Trichoniscus alemannicus* Verhoeff, 1917	+	+	0
*Trichoniscus pusillus* Brandt, 1833	0	+	0
*Trichoniscus* sp.	0	+	+
**Isopod species**	**11**	**13**	**9**
**Isopod specimens**	**3738**	**2690**	**587**
*Brachydesmus superus* Latzel, 1884	+	0	+
*Cylindroiulus caeruleocinctus* (Wood, 1864)	+	0	0
*Cylindroiulus verhoeffi* (Brolemann, 1896)	0	0	+
*Nemasoma varicorne* C. L. Koch, 1847	+	0	0
*Ophyiulus pilosus* (Newport, 1842)	+	+	+!
*Oxidus gracilis* (C. L. Koch, 1847)	0	+	+
*Polydesmus angustus* Latzel, 1884	0	+	0
*Propolydesmus testaceus* (C. L. Koch, 1847)	+	0	0
Chordeumatidae sp.	0	+	0
Craspedosomatidae sp.	0	0	+
indet *+*	+	0	0
**Millipede species**	**5**	**3**	**4**
**Millipede specimens**	**32**	**27**	**46**

Legend: 0: species absent; +: species present; +!: species present at > 25% of sites per city; * taxa identified as a myrmecophilous species (ubiquitous in ant nests); number of specimens were not counted, see text for explanation

We found seven millipede species in Zurich, four in Lugano, and three in Lucerne. *Ophyiulus pilosus* (Newport, 1842) occurred in all three cities, but was dominant in Zurich (62.5%) and Lugano (42.5%), while *Polydesmus angustus* Latzel, 1884 was dominant in Lucerne (85%) (Fig. 1). Two other species [*Brachydesmus superus* Latzel, 1884 and *Oxydus gracilis* (C. L. Koch, 1847)] were exclusively found in Zurich and Lucerne (Table 1).

Millipedes show a different occurrence pattern than isopods, for the latter the differences among the three cities seem to be greater. Overall, 50% of all millipede and isopod species were observed in only one city. The mean number of individuals for isopods and millipedes per site varied substantially in the three cities, i.e. Lucerne [mean 159.7 (SE: 75.3)], Lugano [mean 45 (SE: 12.56)], and Zurich [mean 221.7 (SE: 77.22)]. Sørensen similarity index of species compositions (isopods and millipedes combined) was highest between Zurich and Lucerne (0.67) and lowest between Zurich and Lugano (0.40), with an intermediate value of 0.58 between Lucerne and Lugano.

### Species incidence

Incidences of isopod and millipede species per city were generally low, i.e. below 10% of the total number of traps (Figure 1). For isopods, the most abundant species were also the most frequent ones. The most widespread isopod in Zurich was *Porcellio scaber* (55.6% of 36 traps), while in Lucerne it was *Trachelipus rathkii* (58.8% of 34 traps), and in Lugano *Trachelipus razzautii* (44.4% of 36 traps). Lucerne and Zurich, cities located north of the Alps, shared four isopod species [*Hyloniscus riparius* (C. Koch, 1838), *Porcellio scaber*, *Oniscus asellus* Linnaeus, 1758, and *Trachelipus rathkii*] out of the five most frequently sampled (> 25%). The only millipede with an incidence over 25% (out of 36 traps) was *Ophyiulus pilosus* in Lugano, while the rest of millipede species of our study occurred with incidences < 10% (Figure 1).

There were also examples where higher relative abundances paired with relatively low incidences (<10% occurrence per all sites or per all city) which suggest an aggregated distribution of some species. This was the case for *Trachelipus rathkii* (relative abundance 20%; incidence 6%) and *Armadillidium vulgare* (Latreille, 1804) in Lugano (14%; 6%), and *Armadillidium nasatum* Budde-Lund, 1885 in Zurich (30%; 6%).

## Discussion

### Faunistic results

Next to many taxonomic and faunistic studies on European isopods (Schmalfuss and Wolf-Schwenninger 2002), to the best of our knowledge, there were neither reviews nor faunistic papers published on Swiss Isopoda fauna in the last 100 years since the last overview by Carl (1911). The recent millipede fauna has been summarized by Pedroli-Christen (1993), describing 127 species from the country. Therefore our results are somewhat challenging for interpretation and need to be put in context by considering other studies on the European level.

Our results reveal that the observed cities harbour mostly species widespread in Europe. Regarding isopods, six species are known as widespread in temperate and northern Europe, occupying both urbanized and rural areas: *Armadillidium vulgare*, *Oniscus asellus*, *Philoscia muscorum* (Scopoli, 1763), *Porcellio scaber*, *Trachelipus rathkii*, *Trichoniscus pusillus* Brandt, 1833 (e.g.Hornung et al. 2007, Vilisics et al. 2007). *Orthometopon planum* (Budde-Lund, 1885), a Central European species, is known to dwell in broadleaf forests (Vilisics et al. 2008), but has also been found in the urban fringe of Budapest (Vilisics and Hornung 2009). The only frequent isopod species in Lugano, *Trachelipus razzautii*, seems, instead, to be mainly restricted to northeastern Mediterranean (Schmalfuss 2003).

Among millipedes, seven species are widely distributed across Europe (Enghoff 2010), six of which are known to occur in areas under human influence: *Brachydesmus superus*, *Cylindroiulus caeruleocinctus* (Wood, 1864), *Ophyiulus pilosus*, *Oxidus gracilis*, *Polydesmus angustus*, and *Propolydesmus testaceus* (C. L. Koch, 1847) (Blower 1985, Kime 1990, Pedroli-Christen 1993, Tadler and Thaler 1993).

One isopod (*Trichoniscus alemannicus* Verhoeff, 1917) and one millipede(*Cylindroiulus verhoeffi*) species are known to be restricted to the Alps (Attems 1949, Pedroli-Christen 1993, Schmalfuss, 2003). These cases show that urban areas may support the survival also of native species with a more restricted European distribution.

The isopod *Platyarthrus hoffmannseggii* (Budde-Lund, 1893), a depigmented and blind myrmecophilous species, ubiquitous in ant nests, was frequently sampled in Lucerne. The distribution pattern of such isopods may follow the distribution of their ant hosts.

The intra-European alien (Cochard et al. 2011) *Armadillidium nasatum* is commonly introduced to greenhouses across Europe (Schmalfuss 2003). Reports from various cities show that the species can survive in outdoor habitats, too (e.g. Amsterdam: Berg et al. 2008; Budapest: Vilisics and Hornung 2010). The other non-native species was the millipede *Oxidus gracilis*, a true alien of tropical eastern Asian origin. It has been introduced by human activities into European greenhouses (Blower 1985) and seldom survives outdoors in Europe (Pedroli-Christen 1993).

### Species compositions, abundances and incidence

Our survey in three Swiss cities resulted in a relatively high species richness and abundance of isopods, while millipedes were captured in lower number of species and individuals.

The overall number of millipede species revealed in the three cities is relatively low (8.7%) compared with the known millipede fauna of Switzerland (127 species; Pedroli-Christen 1993). In temperate Europe the average urban species number is between 14 and 26 (Enghoff 1973, Tischler 1980, Korsós 1992, Korsós et al. 2002, Stoev 2004, Bogyó and Korsós 2009, Riedel et al. 2009), despite the fact that only 9 species were recorded in London by Smith et al. (2006).

Reports on urban isopod and millipede fauna show relatively high species richness in temperate cities as compared to known native, local faunas. Such reports are, however, hardly comparable due to differences in sampled habitats, sampling effort and methodology. Pitfall trapping in parks of the city Debrecen (Hungary) resulted to a 19% (14 species) of the known millipede fauna of Hungary (Bogyó and Korsós 2009). A similar method captured ca. 14% (11 species) of the known millipede fauna of the Czech Republic from parks of the city Olomouc (Riedel et al. 2009). Pitfall trapping from parks and nearby rural forested areas in Debrecen and Sorø (Denmark) resulted in Isopoda species (Hornung et al. 2007, Vilisics et al. 2007) comprising 11% of Hungarian and ca. 27% of known Danish Isopoda fauna (Meinertz 1964).

Pooled abundances of isopods and millipedes were well over 1000 individuals in each of the above mentioned studies (90 to 120 operating traps for 6 to 9 months). As the number of millipedes in our study was less than 100 individuals, the question arises why abundances were so low in the three Swiss cities? In his review David (2009) has pinpointed the negative effects of habitat loss and low food quality on millipede assemblages, and demography. Furthermore, management practices altering microhabitats (like coarse woody debris and both litter quantity and quality) has great effect on soil macro-invertebrates, including isopods and millipedes. (e.g. Topp et al. 2006, Kappes et al. 2009). The low activity density values in our studied cities may thus be a result of the intensive management such as mowing and removal of plant litter.

The only alien isopod captured, *Armadillidium nasatum*, also known as the “greenhouse pillbug”, was among the dominating species in Zurich, but it only occurred in 5.7% of the sampling sites (out of the total 36), turning to be one of the rarest species in the city. Similarly, the alien millipede *Oxidus gracilis* (Stoev et al. 2010) showed low incidences, as it appeared in only 3% of the sites in Lucerne, and 12% of Lugano. We assume that these species were either introduced recently in these places or survive in sites which provide special environmental conditions (such as higher annual average temperature), so they may aggregate in high numbers at certain spots of a city, while never establish in others.

The three millipedes (*Ophyiulus pilosus*, *Brachydesmus superus*, *Oxidus gracilis*) occurring in two or more cities are widespread in Europe and occupy rural as well as urban settlements (Blower 1985, Pedroli-Christen 1993, Enghoff 2010). *Ophyiulius pilosus* in Lugano showed the highest incidence of occurrences among the millipedes. We found a somewhat similar trend in isopods, as widely distributed species showed the greatest incidences. More- over, this result is consistent with Kime (1990) and Voigtländer (2011), who suggested that this species might profit from human activities and disturbances.

Pitfall trapping, as the sampling method employed in this study, is a passive sampling method that has been developed to catch specimens active on the soil and litter surface. It has proven to efficiently represent species richness and activity density data of several arthropod groups (e.g. Araneae, Coleoptera) (Standen 2000). As only a fraction of isopod and millipede species (e.g. isopods: family Trichoniscidae, millipedes: *Geoglomeris* sp., *Polyxenus* sp.) actively move in soil/litter and under bark, pitfall trapping is expected to miss ssome less mobile isopod species that are underrepresented in the present samples. Tuf (2003) has captured the isopod *Trichoniscus pusillus* with pitfall traps in good numbers, while Vilisics et al. (2007) reported only a few captured specimens in the midst of a dense *Trichoniscus pusillus* population with the same method. We therefore suppose the pitfall trapping can be useful to assess species diversity and occurrence, while caution remains for abundance data of some species. The data presented here are comparable with other studies that collected isopods and millipedes with the same method. Moreover, because our study sites were sampled during the same periods and with the same method, the data are comparable among sites and yield a fair description of the relative abundances of the different species during these periods of the season in the study area. In case of future faunistic studies, pitfall trapping should be completed with timed hand search (Vilisics et al. 2011). Such a procedure is expected to provide more complete faunistic results, as well as comparable abundance data for soil and litter dwelling organisms such as isopods and millipedes.

### Geographic effects

As with many other invertebrate taxa (e.g. Lepidoptera and Mollusca in IUCN 2011 European Red List), geographic patterns of European isopods and millipedes show a decrease in species richness from southern biodiversity hot-spots to the north. The available literature on millipede and isopod faunas suggests species diversity to decrease roughly by half from Southern Europe to Central Europe, and it further halves towards Fennoscandia (e.g. isopods: Italy: Stoch 2004, Hungary: Hornung et al 2009, Scandinavia: Meinertz 1964); Millipedes: Italy: Stoch 2004, Czech Republic: Tajovský 2001, Scandinavia: Andersson et al. 2005). The Alps are also rich in isopod and millipede species, with cc. 35 endemic isopods (Schmalfuss 2003). Endemic millipedes of Switzerland are known almost exclusively from alpine or subalpine ecosystems (Pedroli-Christen 1993).

Similarities between species compositions hint at the impact of geographical location and distance between cities: the two northern cities (Zurich and Lucerne), which share more species than any other combination of two cities, are only 60 km apart. Lugano is located 170 km south of Lucerne and 210 km south of Zurich, from which it is additionally separated by the Alps. Lugano is already under the influence of Mediterranean climate, which affects the regional flora and fauna.

The common temperate European isopod species (*Trachelipus rathkii*, *Porcellio scaber*, *Oniscus asellus*, *Philoscia muscorum*) are among the most common synanthropic species (i.e. species live near humans and benefit from their association with humans and anthropogenous habitats). Common Mediterranean isopod species are also common in urban sites. Besides *Armadillidium nasatum*, several other species, such as *Agabiformius lentus* (Budde-Lund, 1885) and *Chaetophiloscia cellaria* (Dollfus, 1884), occur further to the north from their natural range and can survive outdoors (e.g. Cochard et al. 2010). Some xerophilic millipedes (e.g. *Brachydesmus superus*) show synanthropic trends in their northern range of distribution as well (Voigtländer 2011). One possible reason for this finding is the so-called “heat island effect” (Andreev 2004) which describes a higher average annual temperature in the city core in comparison with the surrounding areas. Introduced isopod and millipede species often find shelter in private and botanical gardens, but their occurrence is typically aggregated to a restricted area within the city (Vilisics and Hornung 2009, Stoev et al. 2010).

### Biotic homogenization

Biotic homogenization in cities has been described earlier (e.g. McKinney 2006) as an ongoing process characterized by extinction of local faunal elements and dominance of tolerant species, resulting in incresing similarities in species composition among cities. Szlávecz et al. (2008; unpublished) in their presentation at URBIO2008 (Urban Biodiversity & Design conference in Erfurt, Germany), pinpointed a biotic homogenization process on soil dwelling macro-invertebrates (Annelida, Diplopoda, Isopoda) in major European cities. Based on European literature data from the past 50 years (from e.g. Czech Republic, Hungary, Poland, Romania) the list includes 32 species regarded as „homogenizing” in Europe, including 14 isopods and 18 millipede species. Most of the species are widespread throughout temperate Europe, and several of them were introduced to many parts of the world (Blower 1985, Schmalfuss 2003, Enghoff 2010).

In our study we recorded 16 species mentioned in this list, i.e. 11 isopod species (64.7% of the 17 species found in this study) and 5 millipede species (62.5% of 8). The proportion of widespread isopod and millipede species contributing to homogenization was highest in the largest city, Zurich (81.5% of all 25 species), and lowest in the smaller cities, Lugano (70%) and Lucerne (69%).

## Conclusion

Our study showed that urban millipede and isopod assemblages in Switzerland mainly consist of species with wide distribution in Europe. We also showed that cities offer suitable habitats for native and non-native species, with both wide and narrow ecological requirements. Cities under temperate climate showed remarkable differences in their species compositions from the one under Mediterranean influence. This suggests that biogeography plays an important role in shaping isopod and millipede assemblages in the cities.
